# Abdominal trunk muscle weakness and its association with chronic low back pain and risk of falling in older women

**DOI:** 10.1186/s12891-019-2655-4

**Published:** 2019-06-03

**Authors:** Satoshi Kato, Hideki Murakami, Satoru Demura, Katsuhito Yoshioka, Kazuya Shinmura, Noriaki Yokogawa, Takashi Igarashi, Noritaka Yonezawa, Takaki Shimizu, Hiroyuki Tsuchiya

**Affiliations:** Department of Orthopaedic Surgery, Graduate School of Medical Sciences, Kanazawa University, 13-1 Takara-machi, Kanazawa, 920-8641 Japan

**Keywords:** Abdominal trunk muscle, Chronic low back pain, Innovative exercise device, Muscle strength measurement, Older women, Risk of falling

## Abstract

**Background:**

Previous studies have indicated that trunk muscle strength decreases with chronic low back pain, and is associated with poor balance, poor functional performance, and falls in older adults. Strengthening exercises for chronic low back pain are considered the most effective intervention to improve functional outcomes. We developed an innovative exercise device for abdominal trunk muscles that also measures muscle strength. The correlation between muscle weakness, as measured by our device, the presence of chronic low back pain, and decreased physical ability associated with a risk of falling were evaluated in older women.

**Methods:**

Thirty-eight elderly women, who could walk without support during daily activities and attended our outpatient clinic for treatment of chronic low back pain, knee or hip arthritis, or osteoporosis, were included in this study. Anthropometric measurements were performed. Grip power and one-leg standing time with eyes open were measured, and abdominal trunk muscle strength was measured using our device. History of falling in the previous 12 months was noted. Subjects with chronic low back pain (visual analog scale score ≥ 20 mm) for over 3 months were assigned to the low back pain group (*n* = 21). The remaining subjects formed the non-low back pain group (*n* = 17).

**Results:**

Abdominal muscle strength of subjects in the low back pain group, and with history of falling, was significantly lower compared with that of subjects in the non-low back pain group, and in subjects without a history of falling, respectively. There was a moderate positive correlation between abdominal trunk muscle strength and one-leg standing time with eyes open.

**Conclusion:**

We measured abdominal muscle strength in older women with chronic low back pain using our device, and it was significantly lower than that of those without chronic low back pain. Muscle weakness was associated with a history and risk of falling.

## Background

Low back pain (LBP) is a major health problem with great economic and social costs [1]. It is estimated that approximately 80% of the adults have at least one experience of LBP in their lifetime [2, 3]. LBP was found to be the most common type of chronic musculoskeletal pain according to a large-scale survey examining the prevalence and status of chronic musculoskeletal pain in Japan [4]. Trunk muscle weakness has been reported as a risk factor for LBP [5, 6]. Exercise therapy is widely used as a treatment for chronic LBP (CLBP) [7–10]. Some systematic reviews demonstrated that muscle strengthening exercise had a beneficial effect over other interventions in the treatment of CLBP [10, 11]. A substantial proportion of elderly patients with severe CLBP cannot continue the prescribed exercise regimen due to a loss of flexibility and/or deformity in the spine or muscle weakness in the trunk and/or extremities [12, 13].

Locomotive syndrome is a condition of reduced mobility due to impairment of locomotive organs [14]. Progression of this syndrome results in limiting independence in carrying out activities of daily living. A decline in mobility results from one or more disorders of the locomotive structures, including bones, joints, muscles, and nerves [14]. These disorders correlate strongly with future disability, falling, and fractures [15, 16]. Thus, evaluation of physical function and intervention for musculoskeletal disorders are important to maintain quality of life in the elderly. Exercise intervention for locomotive syndrome is effective in improving physical performance, but because most patients are elderly people with significant degenerative diseases of the locomotor organs, caution should be employed when choosing the type and intensity of exercise [17].

**Fig. 1:**
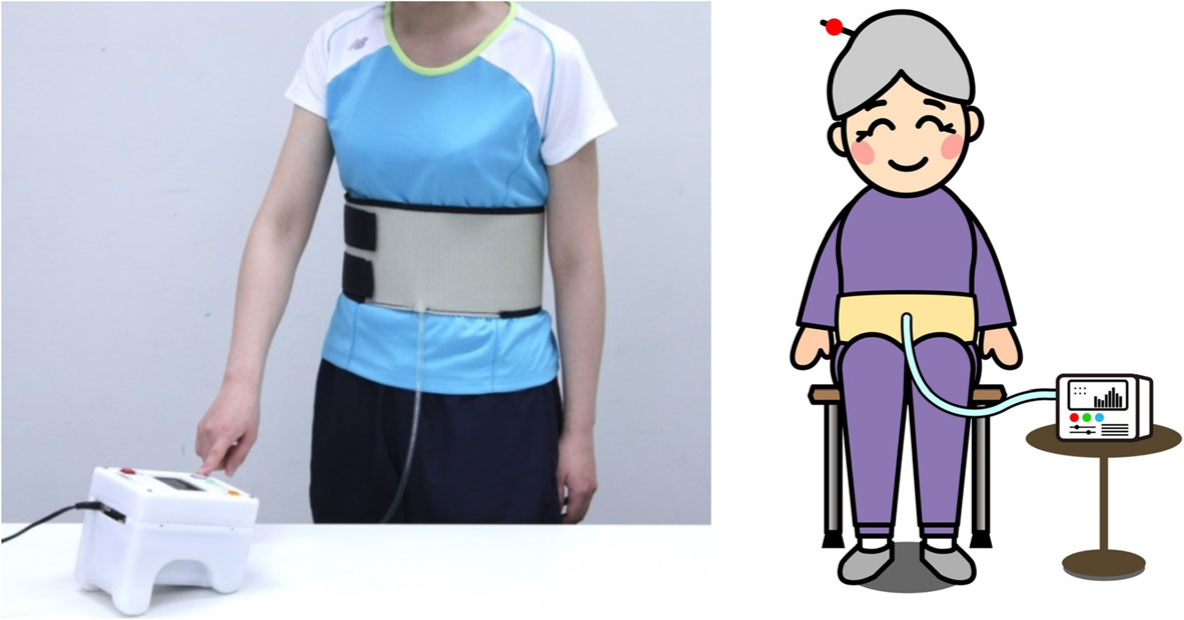
Innovative exercise device for strengthening the abdominal trunk muscle The left image shows a photograph of the device and a subject wearing the inflatable cuff around the abdomen. The right image represents a device-equipped subject in seated position (the image is the authors’ own work)

We developed an innovative exercise device for the abdominal trunk muscles (Fig. 1: trunk muscle exercise device; Nippon Sigmax Co., Ltd., Shinjuku-ku, Tokyo, Japan) [18]. Using this device, subjects can perform strengthening exercises for the abdominal trunk muscles in a sitting position without the need for trunk movement, including movement of the lumbar spine. These exercises are therefore more easily accessible to patients with flexibility loss, deformity of the spine, or severe pain. The device also has a built-in system for measuring abdominal trunk muscle strength, which may reinforce adherence to an exercise program. Our previous studies demonstrated that the device was a reliable tool to measure abdominal trunk muscle strength, and strengthening exercise with the device increased the strength and activated the abdominals, diaphragm, and pelvic-floor muscles to stabilize the spine [19].

In this study, we measured abdominal trunk muscle strength using our device in older women. Our main aim of the study was to investigate the association between muscle weakness and the presence of CLBP. In addition, and as a secondary aim, we studied the relationship between muscle weakness and the risk of falling.

## Methods

### Description of the device

**Fig. 2:**
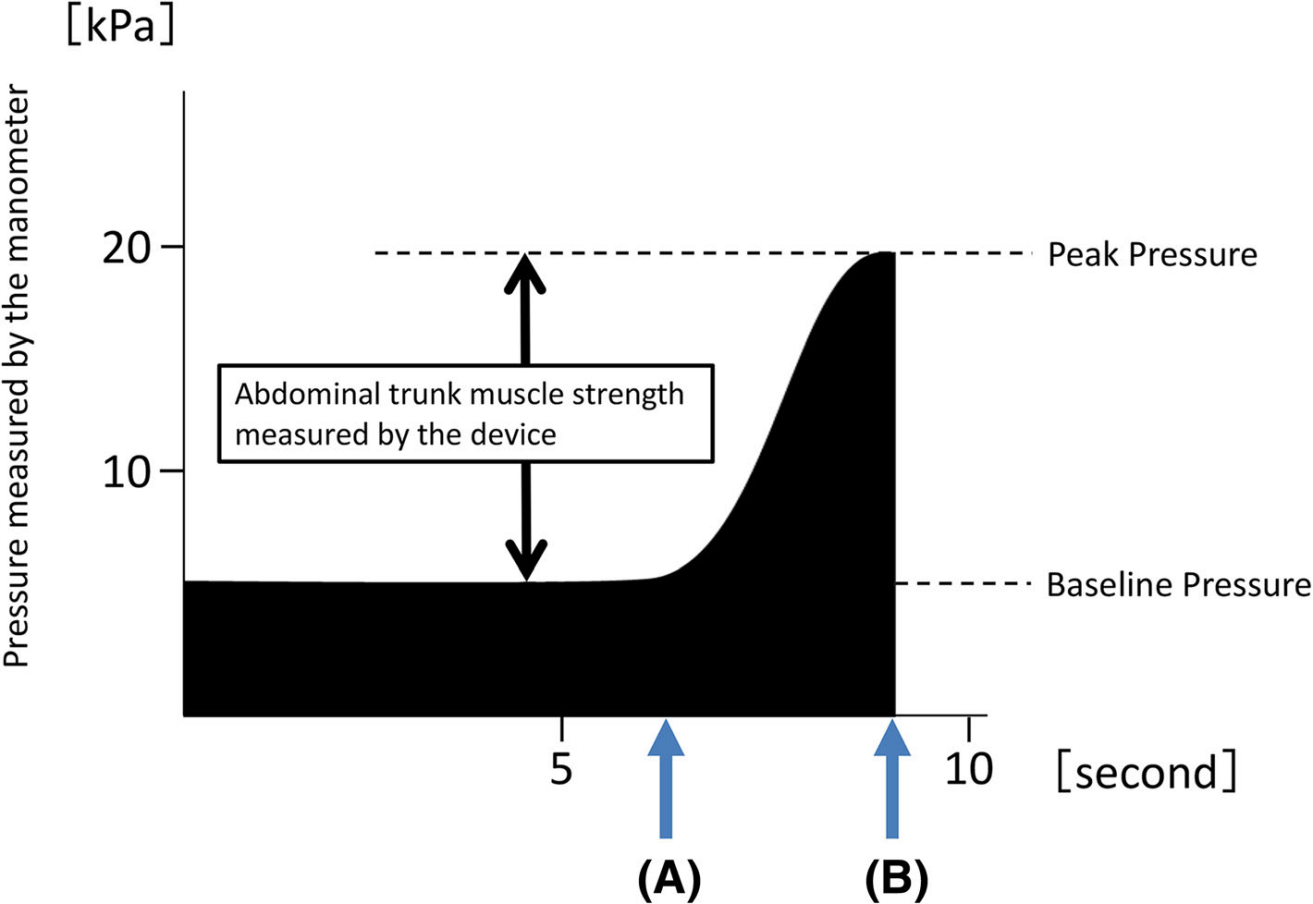
Pressure value time course indicated by the mechanical manometer of the device during measurement of abdominal trunk muscle strength. **a** Indicates the time point when the subject’s abdominal muscles begin to contract against the pressure. **b** Shows the reduction in pressure in the cuff after attainment of peak pressure

As previously described in detail [18], the device has an inflatable cuff and a built-in mechanical manometer to measure pressure. To take a measurement, the cuff placed around the subject’s abdomen is inflated, and an adequate pressure (i.e., the baseline pressure: Fig. 2) is applied to the abdominal wall. Under the baseline pressure, the subject exerts the maximum force by contracting the abdominal muscles. The pressure in the cuff is elevated and reaches a peak (i.e., the peak pressure: Fig. 2). The manometer reports a pressure value that subtracts the baseline pressure from the peak pressure to provide the muscle-strength value. The muscle-strength value was defined in this study as the abdominal trunk muscle strength.

### Subjects

Thirty-eight consecutive elderly women in their 70s or 80s who could walk without support (such as a T-cane or a walker) in their daily activities and agreed to participate in this study were recruited between April and December 2015. They regularly attended out institution as orthopedic outpatients for the treatment of CLBP, knee or hip arthritis, or osteoporosis. Patients who could not complete the following physical examinations conducted in the study due to musculoskeletal or medical disorders were excluded from the study (Table 1). We obtained anthropometric measurements including body height, body weight, body mass index, and girth. We measured each patient’s grip power using a dynamometer, one-leg standing time with eyes open, and abdominal trunk muscle strength using our device. One-leg standing time with eyes open has been shown to be a good predictor of falls in the literature [20–22]. We obtained any history of falling in the previous 12 months and responses to the 25-Question Geriatric Locomotive Function Scale (GLFS-25), which is one of the assessment tools for locomotive syndrome [17]. Twenty-one of the subjects with CLBP for more than 3 months, and a visual analog scale (VAS) score for LBP of ≥20 mm were assigned to the LBP group. For the LBP group, we obtained responses to the Japanese Orthopaedic Association Back Pain Evaluation Questionnaire (JOABPEQ). The JOABPEQ provides specific, yet multidimensional, outcome measures for patients with LBP, including dysfunctions and disabilities caused by the disease, and psychosocial problems resulting from such dysfunctions and disabilities [23]. Seventeen of the subjects without LBP (VAS score = 0 mm) were assigned to the non-LBP group. Subjects with acute LBP or mild CLBP (0 mm < VAS score for CLBP < 20 mm) were excluded from the study. Subjects whose one-leg standing time was shortened owing to their knee or hip pain were excluded from the study. Measurement of one-leg standing time with eyes open was performed once for each of the right and left legs, and with each measurement for a maximum time of 30 s. Subjects were instructed to stand either wearing comfortable shoes or without shoes, with arms placed at their sides. We recorded the measurements with higher values.

**Table 1:** Subject characteristics and exclusion criteria of the study

Characteristic	
No. of subjects	38
Age (years), mean ± SD [range]	77.7 ± 4.2 [70–86]
Height (cm), mean ± SD [range]	148.1 ± 6.0 [137–160]
Weight (kg), mean ± SD [range]	50.5 ± 7.5 [35–67]
Body-mass index (kg/cm^2^), mean ± SD [range]	23.0 ± 3.3 [15.4–30.1]
Girth (cm), mean ± SD	79.7 ± 11.2 [58–110]
Musculoskeletal disorders treated at the outpatient clinic (no. of subjects)	Chronic LBP (21), Knee (22) and hip (3) arthritis, and Osteoporosis (14)
Exclusion criteria of the study	
• They did not agree to participate in this trial.	
• They did not have the ability to walk without support during their daily activities.	
• They could not complete physical examinations conducted in the trial due to their musculoskeletal or medical disorders.	
• They had acute LBP or mild chronic LBP (0 mm < VAS score for chronic LBP < 20 mm).	

*SD* standard deviation, *LBP* low back pain, *VAS* visual analog scale

### Statistical analysis

All data are presented as means and standard deviations. The data were checked for normal distribution with the Shapiro-Wilk test and for homogeneity of the variances with the Levene test. Differences between two groups in continuous variables were examined using the Student t test for parametric data and the Mann-Whitney U test for nonparametric data. Fisher’s exact test was used to determine any association between the presence of CLBP and a history of falling in the past 12 months. Multivariate logistic regression analyses were performed to determine significant factors associated with CLBP and falls. The Pearson correlation coefficient analysis was used to evaluate the correlations between the abdominal trunk muscle strength and age, anthropometric measurements, grip power, one-leg standing time, and GLFS-25 scores. In the LBP group, the Pearson correlation coefficient analysis was used to evaluate the correlations between abdominal trunk muscle strength and VAS scores, as well as the scores in various dimensions of the JOABPEQ. The level of statistical significance was set to 0.05. SPSS software version 19.0 for Windows (SPSS Inc., Chicago, Illinois, USA) was used for all statistical analyses.

## Results

None of the participants experienced pain or discomfort during the measurement of abdominal trunk muscle strength using our device, or during grip power measurement or the one-leg standing time with eyes open test. In the univariate analysis, there were no differences between the LBP and non-LBP groups with regards to age, anthropometric measurements, grip power, one-leg standing time, or history of falling (Table 2). However, abdominal trunk muscle strength as measured by our device was significantly lower in the LBP group than in the non-LBP group (5.1 ± 2.4 kPa versus 7.1 ± 3.2 kPa, *P* <  0.05; Table 2). Furthermore, GLFS-25 scores were significantly higher in the LBP group (Table 2). The multivariate logistic regression analysis showed that abdominal trunk muscle strength (*P* = 0.03; odds ratio, 0.71) and GLFS-25 scores (*P* = 0.03; odds ratio, 1.10) were independently associated with CLBP (Table 3).

**Table 2:** Univariate analysis of characteristics between the LBP group and Non-LBP groups

Characteristics	LBP group	Non-LBP group	*p* value
No. of patients	21	17	
Age (years), mean ± SD	77.4 ± 4.2	78.1 ± 4.4	0.67
Height (cm), mean ± SD	147.5 ± 6.2	148.8 ± 5.7	0.50
Weight (kg), mean ± SD	50.7 ± 7.8	50.2 ± 7.4	0.85
Body-mass index (kg/cm^2^), mean ± SD	23.3 ± 3.7	22.7 ± 2.9	0.53
Girth (cm), mean ± SD	81.7 ± 11.6	77.3 ± 10.6	0.24
Abdominal trunk muscle strength measured by the device (kPa), mean ± SD	5.1 ± 2.4	7.1 ± 3.2	0.03
Grip power (kg), mean ± SD	17.5 ± 4.0	19.1 ± 3.9	0.23
One-leg standing time with eyes open (sec), mean ± SD	12.1 ± 7.7	16.8 ± 10.2	0.11
GLFS-25 Scores, mean ± SD	21.9 ± 12.2	13.4 ± 10.2	0.03
History of falling in the previous 12 months, No. (%)	6 (28.6%)	4 (23.5%)	0.51

*SD* standard deviation, *LBP* low back pain

**Table 3:** Multivariate logistic regression analysis of factors correlated with chronic LBP

Factors	OR	95% CI	*p* value
Abdominal trunk muscle strength (kPa)	0.707	0.515–0.971	0.032
GLFS-25 Scores	1.098	1.009–1.194	0.030

*LBP* low back pain, *OR* odds ratio, *CI* confidence interval, *GLFS-25* the 25-Question Geriatric Locomotive Function Scale

Of 39 subjects, 10 had a history of falling in the past 12 months (fall group), while the remainder of the patients (*n* = 28) did not (non-fall group). In the univariate analysis, there were no significant differences between the fall and non-fall groups with regards to age, body height, body-mass index, girth, one-leg standing time, or GLFS-25 score (Table 4). However, abdominal trunk muscle strength as measured by our device, grip power, and body weight were significantly lower in the fall group (Table 4). The multivariate logistic regression analysis showed that abdominal trunk muscle strength (*P* = 0.01; odds ratio, 0.45) and body weight (*P* = 0.03; odds ratio, 0.83) were independently associated with a history of falling in the previous 12 months (Table 5).

**Table 4:** Univariate analysis of characteristics between the Fall and non-Fall groups

Characteristics	Fall group	Non-Fall group	*p* value
No. of patients	10	28	
Age (years), mean ± SD	79.5 ± 4.1	77.1 ± 4.2	0.12
Height (cm), mean ± SD	146.2 ± 6.6	148.8 ± 5.7	0.25
Weight (kg), mean ± SD	46.4 ± 8.4	52.0 ± 6.8	0.04
Body-mass index (kg/cm^2^), mean ± SD	21.7 ± 3.5	23.5 ± 3.1	0.13
Girth (cm), mean ± SD	77.6 ± 12.0	80.5 ± 11.1	0.50
Abdominal trunk muscle strength measured by the device (kPa), mean ± SD	3.8 ± 1.7	6.8 ± 2.9	< 0.01
Grip power (kg), mean ± SD	15.4 ± 2.6	19.3 ± 3.9	< 0.01
One-leg standing time with eyes open (sec), mean ± SD	12.6 ± 9.6	14.8 ± 9.0	0.52
GLFS-25 Scores, mean ± SD	20.0 ± 15.6	17.4 ± 10.7	0.57
CLBP for ≥3 months (VAS ≥20), No. (%)	6 (60.0%)	15 (53.6%)	0.51

*SD* standard deviation, *GLFS-25* the 25-Question Geriatric Locomotive Function Scale, *CLBP* chronic low back pain, *VAS* visual analog scale

**Table 5:** Multivariate logistic regression analysis of factors correlated with a history of falling in the previous 12 months

Factors	OR	95% CI	*p* value
Weight (kg)	0.833	0.707–0.982	0.029
Abdominal trunk muscle strength (kPa)	0.446	0.235–0.846	0.014

*OR* odds ratio, *CI* confidence interval

**Fig. 3:**
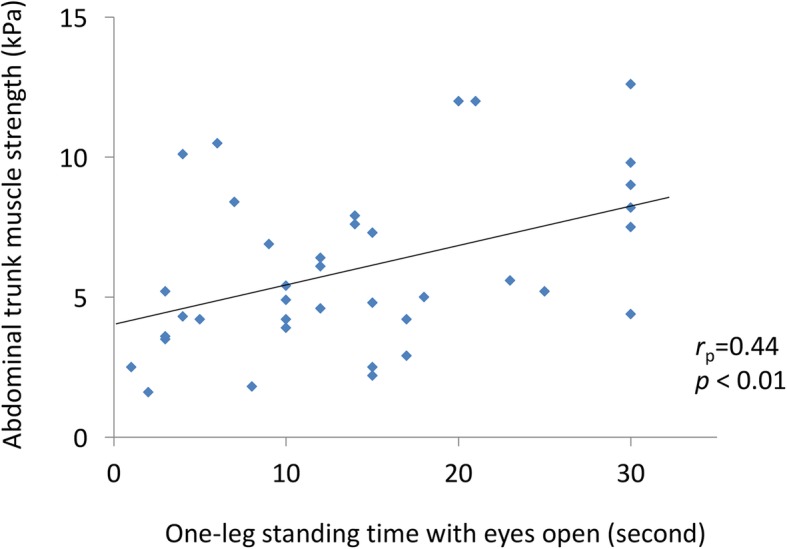
Correlation between muscle strength values as measured by our device and one-leg standing time with eyes open

There was a moderate positive correlation between abdominal trunk muscle strength and one-leg standing time with eyes open (r_p_ = 0.44, *P* <  0.01; Table 6, Fig. 3). However, there was no correlation between abdominal trunk muscle strength and other items evaluated (Table 6). There was no correlation between abdominal trunk muscle strength and VAS score for CLBP, or scores in the various dimensions of the JOABPEQ in the LBP group. The correlation between muscle strength and pain-related disorder scores on the JOABPEQ was nearly significant (r_p_ = 0.40, *P* = 0.07; Table 7).

**Table 6:** Correlation with the Abdominal trunk muscle strength measured by the device in the all 38 participants

	Mean ± SD	Correlations with abdominal trunk muscle strength	
		*R*_*p*_ value	*P* value
Abdominal trunk muscle strength measured by the device (kPa)	6.0 ± 4.0	–	–
Age (years)	77.7 ± 4.2	−0.23	0.16
Height (cm)	148.1 ± 4.8	− 0.11	0.51
Weight (kg)	50.0 ± 7.5	−0.12	0.48
Body-mass index (kg/cm^2^)	23.0 ± 3.3	−0.05	0.77
Girth (cm)	84.4 ± 6.3	−0.23	0.17
Grip power (kg)	79.7 ± 11.2	0.25	0.13
One-leg standing time with eyes open (sec)	14.2 ± 9.1	0.44	< 0.01
GLFS-25 Scores	18.1 ± 12.0	−0.01	0.95

*SD* standard deviation, *GLFS-25* the 25-Question Geriatric Locomotive Function Scale

**Table 7:** Correlation with the Abdominal trunk muscle strength measured by the device in the 21 patients of the LBP group

	Mean ± SD	Correlations with abdominal trunk muscle strength	
		*R*_*p*_ value	*P* value
Abdominal trunk muscle strength measured by the device (kPa)	5.1 ± 2.4	–	–
VAS for CLBP	45.6 ± 15.4	−0.15	0.52
The dimensions of JOABPEQ			
Scores for pain-related disorders	64.6 ± 27.6	0.40	0.07
Scores for lumbar spine dysfunction	68.1 ± 15.7	0.12	0.60
Scores for gait disturbance	62.9 ± 26.5	0.16	0.50
Scores for social life function	62.8 ± 14.1	−0.35	0.13
Scores for psychological disorders	52.6 ± 14.0	− 0.19	0.42

*SD* standard deviation, *VAS* visual analog scale, *CLBP* chronic low back pain, *JOABPEQ* the Japanese Orthopaedic Association Back Pain Evaluation Questionnaire

## Discussion

This study sought to examine whether abdominal trunk muscle strength, measured using a novel device, was associated with CLBP or risk of falling in elderly women. Our results demonstrated that abdominal muscle strength of subjects with CLBP was significantly lower than that of subjects without CLBP. Furthermore, muscle strength weakness was associated with a history and risk of falling. A history of falling in the past 12 months and one-leg standing time with eyes open have been reported to be significant risk factors for falling in the elderly [22, 24, 25]. Locomotive syndrome assessment scores were higher in subjects with CLBP in our study.

Several studies have indicated that patients with LBP have significantly lower abdominal muscle strength than asymptomatic patients [26–28]. A previous study demonstrated that muscle strength, as measured using our exercise device, was correlated with trunk flexor strength, including the abdominal rectus and abdominal oblique located in the anterolateral aspect of the abdomen [18]. The diaphragm, abdominal rectus, external and internal oblique, transverse abdominal, and levator ani muscles were significantly activated while strengthening using the device [19].

The abdominal core can be described as a muscular box with the abdominals in the front and sides, paraspinals in the back, diaphragm as the roof, and pelvic floor muscles as the bottom [29]. The abdominal contraction maneuver under a pressure produced by the device is like abdominal bracing and creates the coordinated contraction of the deep and superficial core muscles at the anterolateral aspect, the top and bottom of the “muscular box” of the core [19]. The strength of abdominal trunk muscles, as measured by the device, is generated by the contraction of these core muscles to increase intra-abdominal pressure and spinal stability. The results of this study indicated that abdominal trunk muscle weakness measured by the device was associated with CLBP in older women. The muscle strengthening exercises undertaken with the device may be a viable option for the treatment of patients with CLBP, especially in older adults with lowered physical ability.

One systematic review reported that weak trunk muscle strength was correlated with poor balance and functional performance, and more falls in older adults [30]. Granacher et al. demonstrated that core instability strength training mitigated trunk muscle strength, dynamic balance, and functional mobility in older adults [31]. Consistent with these studies, our results have indicated that weak abdominal trunk muscle strength, as measured by our device, was associated with decreased static balance and function, and increased risk of falling, regardless of CLBP.

The limitations of the present study include its small sample size and the inclusion of a significant portion of participants with other locomotive organ disorders, which may have affected the results. There was no correlation between abdominal trunk muscle strength and GLFS-25 scores because other locomotive organ disorders may have influenced the scores. The causes of CLBP in the study subjects were multifactorial and they were not examined. Muscle strength of the back and lower extremities was not measured, and spinal alignment was not analyzed. The correlation between abdominal trunk muscle strength and the strength of these muscles or spinal deformities was not evaluated in this study. In addition, extrinsic and environmental risk factors for falling were not obtained or evaluated in this study. Further studies with larger cohorts and that include men, are required to confirm the results of our study. Future studies involving older patients with CLBP and deteriorated physical ability are also needed to validate the efficacy of the device for the treatment of patients with CLBP and locomotive syndrome.

Despite its limitations, this study showed that the abdominal muscle strength of older patients with CLBP was significantly lower than that of subjects without CLBP, and that muscle strength weakness was associated with a history and risk of falling. Our device is a viable option for measuring abdominal muscle strength as a factor contributing to physical function.

## Conclusion

We measured abdominal muscle strength in older women with CLBP using a novel device, and found that it was significantly lower than that of those without CLBP. Muscle weakness was associated with a history and risk of falling. Further studies are needed to validate the efficacy of our device for the treatment of CLBP and locomotive syndrome.

## Data Availability

The datasets during and/or analyzed during the current study are available from the corresponding author on reasonable request.

## Abbreviations

CLBP: Chronic low back pain
GLFS-25: 25-Question Geriatric Locomotive Function Scale
JOABPEQ: Japanese Orthopaedic Association Back Pain Evaluation Questionnaire
LBP: Low back pain
VAS: Visual analog scale
